# Safety Assessment of Methanol Extract of *Melastoma malabathricum* L. Leaves following the Subacute and Subchronic Oral Consumptions in Rats and Its Cytotoxic Effect against the HT29 Cancer Cell Line

**DOI:** 10.1155/2019/5207958

**Published:** 2019-11-26

**Authors:** N. E. Kamsani, Z. A. Zakaria, N. L. Md Nasir, N. Mohtarrudin, N. B. Mohamad Alitheen

**Affiliations:** ^1^Department of Biomedical Science, Faculty of Medicine and Health Sciences, Universiti Putra Malaysia, 43400 UPM Serdang, Selangor Darul Ehsan, Malaysia; ^2^Department of Pathology, Faculty of Medicine and Health Sciences, Universiti Putra Malaysia, 43400 UPM Serdang, Selangor Darul Ehsan, Malaysia; ^3^Department of Cell and Molecular Biology, Faculty of Biotechnology and Biomolecular Sciences, Universiti Putra Malaysia, 43400 UPM Serdang, Selangor Darul Ehsan, Malaysia

## Abstract

Methanol extract of *Melastoma malabathricum* (MEMM) has been traditionally used by the Malay to treat various ailments. In an attempt to develop the plant as an herbal product, MEMM was subjected to the subacute and subchronic toxicity and cytotoxicity studies. On the one hand, the subacute study was performed on three groups of male and three groups of female rats (*n* = 6), which were orally administered with 8% Tween 80 (vehicle control group) or MEMM (500 and 1000 mg/kg) daily for 28 days, respectively. On the other hand, the subchronic study was performed on four groups of rats (*n* = 6), which were orally administered with 8% Tween 80 (vehicle control group) or MEMM (50, 250, and 500 mg/kg) daily for 90 days, respectively. In the *in vitro* study, the cytotoxic effect of MEMM against the HT29 colon cancer cell line was assessed using the MTT assay. MEMM was also subjected to the UHPLC-ESI-HRMS analysis. The results demonstrated that MEMM administration did not cause any mortality, irregularity of behaviour, modification in body weight, as well as food and water intake following the subacute and subchronic oral treatment. There were no significant differences observed in haematological parameters between treatment and control groups in both studies, respectively. The *in vitro* study demonstrated that MEMM exerts a cytotoxic effect against the HT29 colon cancer cell line when observed under the inverted and phase-contrast microscope and confirmed by the acridine orange/propidium iodide (AOPI) staining. The UHPLC-ESI-HRMS analysis of MEMM demonstrated the occurrence of several compounds including quercetin, *p*-coumaric acid, procyanidin A, and epigallocatechin. In conclusion, *M. malabathricum* leaves are safe for oral consumption either at the subacute or subchronic levels and possess cytotoxic action against the HT29 colon cancer cells possibly due to the synergistic action of several flavonoid-based compounds.

## 1. Introduction

*Melastoma malabathricum* (family Melastomaceae) has been applied in various folklore medicines to heal diverse forms of maladies [1–3]. Scientifically, various reports on *M. malabathricum* have been published and extensively reviewed by Joffry et al. [4]. With regard to the methanol extract of *M. malabathricum* leaves (MEMM) toxic effect, only the acute toxicity study has been carried out on MEMM using the OECD Guideline No. 423 [5]. Interestingly, MEMM was reported to show no toxic effect at the dose of 5000 mg/kg when given orally [5]. Meanwhile, with regard to the cytotoxic effect of *M. malabathricum*, MEMM has been reported to show cytotoxic activities against several murine (i.e., 3LL (Lewis lung carcinoma cells) and L1210 (leukemic cells)) and human (i.e., K562 (chronic myeloid leukaemia), DU145 (prostatic adenocarcinoma), MCF-7 (mammal carcinoma), and U251 (glioblastoma)) cancer lines [6], but not Vero cell line (African green monkey, *Cercopitheus aethiops* kidney cells) and mouse fibroblast cell line (L929) [7], respectively.

Along the course of developing a potent natural-based product, toxicity or the adverse effects of the plant extract on living organisms has been one of the major issues. Although *M. malabathricum* has been given the herbal status in Malaysia and several scientific studies have confirmed its medicinal values, further studies need to be carried out to establish its toxicity profile. In addition, despite the cytotoxic reports of MEMM described above, no attempt has been made to investigate the cytotoxic effect of MEMM against the HT29 colon cancer cell line. Taking into account that (i) MEMM was reported to be safe at 5000 mg/kg when assessed using the acute toxicity model MEMM and (ii) MEMM was cytotoxic only against 3LL, L1210, and U251 cancer cell lines with no report against HT29 colon cancer cell line, the present study was designed to determine the toxic effect of MEMM following its subacute and subchronic oral exposure for 28 or 90 days, respectively, and to evaluate the cytotoxic activity of MEMM against the HT29 cancer cell line.

## 2. Materials and Methods

### 2.1. Sample Collection and Preparation of Crude Extract

The leaves of *M. malabathricum* were collected between June and July 2013 from its natural habitat around Serdang, Selangor, Malaysia, based on the voucher specimen (SK 2199/13) deposited earlier in the herbarium of Institute of Bioscience, Universiti Putra Malaysia.

The crude extract of *M. malabathricum* was prepared as stated by Mamat et al. [8]. In brief, the dried leaves in powder form (200 g) were soaked in 4000 mL methanol for 72 h and this procedure was performed three times to obtain the supernatant. The supernatant was then evaporated at 40°C under reduced pressure to acquire the crude methanol extract (MEMM). The extract was left in the oven at 40°C to allow the solvent residue to dry and occasionally weighed until a constant weight was obtained.

### 2.2. In Vivo Study

#### 2.2.1. Experimental Animals

Sprague-Dawley rats (4 weeks of age; weighed between 120 and 150 g) were purchased from the Faculty of Veterinary Medicine, UPM, and acclimatized in the animal house of the Faculty of Medicine and Health Sciences (FMHS), UPM, under control temperature (22 ± 2°C), 70–80% humidity with 12 h light/dark cycle. The rats were allowed access to basal diet and water ad libitum and monitored according to the guidelines accepted by the Institutional Animal Care and Use Committee (IACUC), FMHS, UPM [8]. Ethics approval for animal care and use was approved by the IACUC (IACUC no: UPM/IACUC/AUP-R007/2014).

#### 2.2.2. Preparation and Administration of Different Dosages of MEMM

MEMM was dissolved in 8% Tween 80 to the required dose using a sonicator at 40°C for 5–10 min. Animals were administered orally with the freshly prepared extract or vehicle daily according to their body weight [9].

#### 2.2.3. Subacute Toxicity Study

Eighteen (18) male and eighteen (18) female rats were each randomly allocated into three groups (*n* = 6) and treated via oral gavage daily for 28 days with 8% Tween 80, 500 mg/kg MEMM, or 1000 mg/kg MEMM. Body weight was recorded weekly and on the 28th day prior to the termination of the experiment. The subacute toxicity study was performed according to the OECD Test Guideline 407 [10].

#### 2.2.4. Subchronic Toxicity Study

Twenty-four male rats were randomly allocated into four groups (*n* = 6) and treated via oral gavage daily for 90 days with 8% Tween 80, 50 mg/kg MEMM, 250 mg/kg MEMM, or 500 mg/kg MEMM. Body weight was recorded weekly and on the 90th day prior to the termination of the experiment. The subchronic toxicity study was performed according to the OECD Test Guideline 408 [11].

#### 2.2.5. Sample Collection

Depending on the model of toxicity studies, the rats were weighed and then anesthetized with ketamine (100 mg/kg; intramuscular (i.m.)) and xylazine (16 mg/kg; i.m.) on the respective 28^th^ or 90^th^ day prior to the collection of blood and tissues [12]. The whole blood was obtained through cardiac puncture and kept in the labeled plain and EDTA-containing vacutainer tubes (BD Plymouth, UK). Blood in the EDTA vacutainers was kept at 4°C, while blood in the plain (non-EDTA-containing) vacutainers was subjected to the centrifugation procedure (at 1500 g for 3 min) to obtain the serum, which was then kept at −20°C. Following the blood collection, the rats were sacrificed by cervical dislocation and then dissected to collect the important organs, namely, liver, spleen, kidneys, stomach, heart, and lungs. Each organ was rinsed with normal saline and their weights were then recorded before being fixed in 10% buffered formalin for further histopathological study.

#### 2.2.6. Haematological and Biochemical Analysis

The toxic outcome of subacute or subchronic oral administration of MEMM was evaluated using the samples collected as described above. The whole blood in the EDTA-containing vacutainers was processed within 24 h and then subjected to the haematological analysis using the automated analyser (Coulter STKS, Beckman) to yield information on several haematological parameters (i.e., total red blood cell (RBC) count, haemoglobin (Hb), mean corpuscular haemoglobin concentration (MCHC), mean corpuscular volume (MCV), packed cell volume (PCV), total white blood cell (WBC) count, neutrophils, monocytes, lymphocytes, and eosinophils).

The serum, which was earlier kept at −20°C, was thawed at 25°C and treated within two days following the blood collection. Biochemical analysis was performed using an automated biochemical analyser (Hitachi 902, Japan) for several biochemical parameters (i.e., alanine aminotransferase (ALT), alkaline phosphatase (ALP), aspartate aminotransferase (AST), creatinine (Crea), urea, and total bilirubin (TBil)). The control group mean values were used as the baseline for comparison with treatment groups.

#### 2.2.7. Histopathological Study

The whole rat organs, collected following the subacute and subchronic studies and kept in 10% buffered formalin, were sectioned and prepared as previously described [13]. Sections were stained with Haematoxylin and Eosin (H&E) and then microscopically examined for pathological changes at 10x, 20x, and 40x magnifications.

### 2.3. In Vitro Study

#### 2.3.1. Cell Line Cultivation

Normal mouse fibroblast (3T3) and human colon cancer (HT29) cell lines were procured from the American Type Culture Collection (ATCC). Cells were grown and maintained in Roswell Park Memorial Institute-1640 (RPMI) media added with 10% fetal bovine serum (FBS) and 1% penicillin-streptomycin (Pen-Strep). Cells were cultured at humidified atmosphere 5% carbon dioxide (CO_2_) at 37°C in an incubator. Trypsin-EDTA was then used to detach the confluent cells at 80% confluency. The cells were then stained with trypan blue before the cell number, and viability was determined using a haemocytometer [14].

#### 2.3.2. Cell Proliferation Assay

1 × 10^5^ cells (3T3 or HT29) were seeded in a 96-well plate. Upon 24 h of incubation at 37°C with 5% CO_2_ atmosphere, the media were discarded and replaced with fresh complete media (10% FBS and 1% Pen-Strep) containing MEMM dried extract. The dried extract was diluted in dimethyl sulfoxide (DMSO) to prepare the starting concentration of 200 *μ*g/mL, which was then serially diluted to the lowest concentration of 0.156 *μ*g/mL. Negative control was not treated with MEMM. After 72 h of incubation, 20 *μ*L of 3-(4,5-dimethylthiazol-2-yl)-2,5-diphenyltetrazolium bromide (MTT) in phosphate buffer saline (PBS) was added to each well. The plate was incubated again for 3 to 4 h. Next, the medium from each well was discarded, and 100 *μ*L of DMSO was added, mixed thoroughly for 5 min to form purple formazan. The plate was then read using the ELISA reader. A graph of cell viability against concentration was plotted. Inhibitory concentration (IC_50_) can be defined as the concentration of MEMM that caused approximately 50% of cell death [14].

#### 2.3.3. Inverted Microscope Study

HT29 cells were seeded at 1 × 10^5^ cells in a 6-well plate and incubated overnight at 37°C with 5% CO_2_ atmosphere. After incubation, the media was discarded and substituted with fresh complete media containing MEMM. After 72 h of incubation, morphological changes of the cells were examined by using an inverted microscope at 10x, 20x, and 40x actual magnification [15].

#### 2.3.4. Phase-Contrast Examination

HT29 cells were seeded in a 6-well plate with completed media and incubated at 37°C with 5% CO_2_ atmosphere. The media was discarded and substituted with fresh complete media containing MEMM on the next day. After 72 h of incubation, the cellular structure in the bright field was examined by phase contrast using a light microscope at 10x, 20x, and 40x actual magnification [15].

#### 2.3.5. Acridine Orange/Propidium Iodide (AOPI) Staining

Approximately, 1 × 10^5^ of HT29 cells were seeded in a 6-well plate and incubated for 24 h at 37°C with 5% CO_2_ atmosphere. The media was discarded and substituted with fresh complete media containing MEMM and incubated again for 72 h. After incubation, the cells were trypsinized with trypsin-EDTA and washed with PBS. 10 *μ*L of cells from each well were put on a glass slide and mixed with 10 *μ*l of acridine orange (AO) (50 *μ*g/mL) and 10 *μ*l of propidium iodide (PI) (50 *μ*g/mL). The nucleus of viable and dead cells was viewed under a fluorescence microscope for qualitative and quantitative approaches [16].

#### 2.3.6. High-Resolution UPLC-ESI-HRMS Analysis of MEMM

Chromatography separation of MEMM was performed on the Dionex Ultimate 3000 RS UHPLC system comprising a UHPLC pump, an auto sample operating at 4°C, and Exactive Orbitrap mass spectrometer with a heated electrospray ionization probe operating in the negative ionization mode (Thermo Fisher Scientific, San Jose, CA). In brief, reverse separations were carried out using the RP Max column (250 × 4.6 mm, particle size 4.0 *μ*m; Synergi) maintained at 40°C and eluted at a flow rate of 0.3 mL/min with 25 min gradient of 10–50% of 0.1% acidic acetonitrile in 0.1% aqueous formic acid. The conditions were set as follows: sheath gas at 15 (arbitrary units), aux at 20 and sweep gas at 5 (arbitrary units), spray voltage at 3.0 kV, capillary temperature at 350°C, and s-lens RF level at 55 V. The mass range was from 100 to 1500 amu with a resolution of 17,000, FTMS AGC target at 2*e*5, FT-MS/MS AGC target at 1*e*5, isolation width of 1.5 amu, maximum ion injection time of 500 ms, and the normalization collision energy at 35%.

#### 2.3.7. Statistical Analyses

The results obtained were statistically analysed (Graph Pad Prism version 5.02) using the one-way analysis of variance (ANOVA) followed by the Dunnett post hoc test. Data were expressed as means ± standard error mean (SEM) with *P* < 0.05 as the limit of significance.

## 3. Results

### 3.1. In Vivo Subacute and Subchronic Toxicity Studies

In the present study, the subacute and subchronic toxicities of MEMM were assessed in rats after the daily oral consumption of MEMM for 28 and 90 days, respectively. The dosages used for the subacute toxicity study was 500 and 1000 mg/kg/day, while for the subchronic toxicity study, the dosages used were 50, 250, and 500 mg/kg/day.

### 3.2. Findings on the Physical Signs, Body Weight, and Food and Water Consumption

All tested groups of rats were found to be equally healthy throughout both toxicity studies, wherein no signs of changes in behaviour were observed. Interestingly, the administration of MEMM for 28 or 90 days also did not cause any clinical signs of toxicity or mortality in rats. Although weight gain was detected in all rats administered with MEMM throughout the experimental periods, a comparison between the MEMM-treated groups against the control group showed insignificant (*P* > 0.05) changes in the weight gain at their respective interval (data not shown). In addition, insignificant (*P* > 0.05) changes in food and water consumption were seen when the MEMM-treated rats were compared to the normal control rats (data not shown).

### 3.3. Relative Organ Weight and Macroscopic Findings

The relative organ weight of Sprague-Dawley rats following the subacute and subchronic toxicity studies is shown in Tables 1 and 2, respectively. There were insignificant (*P* > 0.05) changes in the liver weight/body weight ratio for all the collected organs, namely, liver, kidneys, heart, spleen, lungs, or stomach of MEMM-treated rats in comparison to the normal control rats. Further macroscopic assessments of the harvested organs showed that the MEMM pretreatment did not change the colour or induce hypertrophy of those organs when compared against the respective organ of the normal control group (data not shown).

### 3.4. Haematological and Biochemical Findings

To support the above observations, haematological and biochemical analyses were also performed on the blood, liver, and urine collected from the rats. Analysis of the haematological parameters of MEMM-treated groups also showed insignificant (*P* > 0.05) difference when compared to the normal control group for both the subacute (Table 3) and subchronic (Table 4) studies. A normal haematological profile was recorded in MEMM-treated groups in comparison to the normal control group for both toxicity studies.

On the contrary, biochemical analysis of the hepatic and renal function parameters demonstrated that MEMM treatment caused insignificant (*P* > 0.05) changes in all parameters evaluated in both the toxicity studies (Table 5), except for the hepatic function parameters of the subchronic toxicity study, whereby a significant (*P* < 0.05) increase in the level of ALP was observed only in the groups pretreated with 250 and 500 mg/kg MEMM in comparison to the normal control group (Table 6).

### 3.5. Histopathology and Microscopic Findings

**Figure 1:**
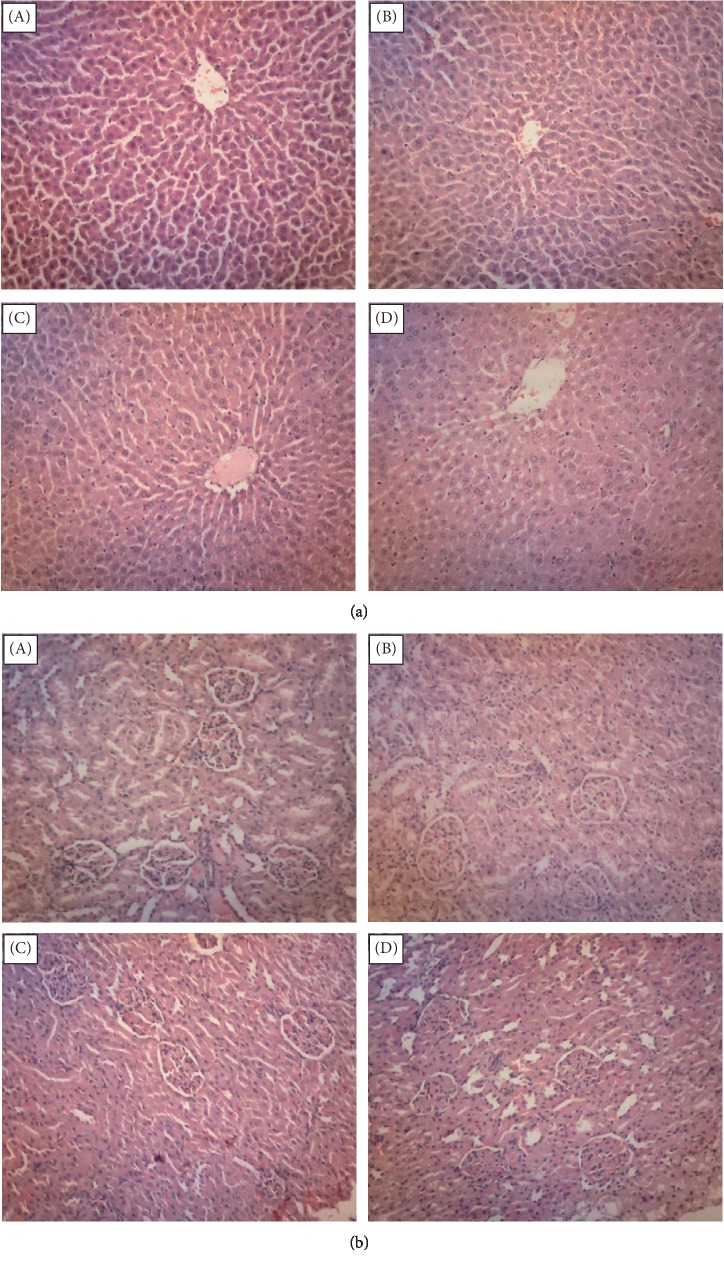
(a) Micrographs of liver section showing normal architecture following treatment with (A) Tween 80 (normal control), (B) 50 mg/kg MEMM, (C) 250 mg/kg MEMM, and (D) 500 mg/kg MEMM (H&E, 400x). (b) Micrographs of kidney section showing normal architecture following treatment with (A) Tween 80 (normal control), (B) 50 mg/kg of MEMM, (C) 250 mg/kg of MEMM, and (D) 500 mg/kg of MEMM (H&E 400x).

Further support on the above observations was obtained microscopically wherein all harvested organs pretreated with MEMM in the subacute and subchronic toxicity studies demonstrated no signs of toxicity when compared to the respective normal control group. This assumption is made based on the indication that there is no change in the architecture of cells when observed under the light microscope at numerous magnification powers. No pathological signs were documented in the histological analysis of the vital organs of the control group. Thus, not all figures of the collected organs were presented in the manuscript. Only micrographs of selected organs (i.e., liver and kidney) harvested from rats following the subchronic toxicity study were shown (Figures 1(a) and 1(b), respectively).

### 3.6. In Vitro Cytotoxic Study

#### 3.6.1. Cytotoxicity Effect against HT29 Cells

The cytotoxic potential of MEMM, at the highest concentration of 200 *μ*g/ml, was evaluated against HT29 cells using the MTT assay. The extract was serially diluted to produce a concentration range of 0.156–200 *μ*g/ml and then subjected to the MTT assay.

#### 3.6.2. Antiproliferative Effect against HT29 Cell Line

Treatment with MEMM had promoted antiproliferation of the HT29 cells. After 72 h, cell proliferation was decreased to 50% compared to the untreated HT29 cells. The IC^50^ obtained for MEMM was approximately 100 *μ*g/mL.

#### 3.6.3. Inverted Microscope Observation

**Figure 2:**
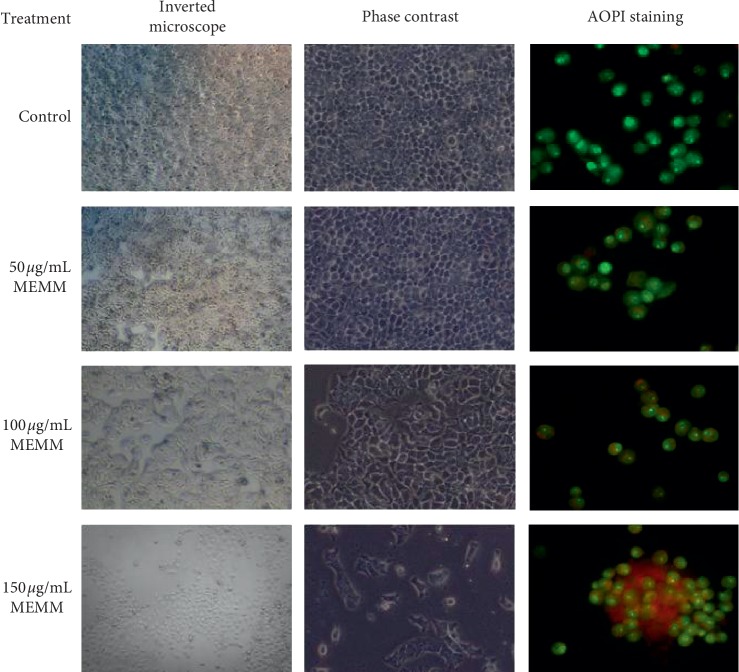
Images showing the effect of MEMM at the concentration of 50, 100, and 150 *μ*g/mL on human colon cancer cell line (HT29) captured from inverted microscope, phase contrast, and AOPI staining after 72 h.

After incubation with MEMM (50, 100, and 150 *μ*g/mL), morphological modifications in HT29 cells were seen at 100 and 150 *μ*g/mL and compared to the control untreated cells (Figure 2). Untreated HT29 cells were observed with normal morphology while those treated with 100 and 150 *μ*g/mL MEMM exerted retraction and rounding of cells with some sensitive cells detached from the surface.

#### 3.6.4. Phase-Contrast Examination

After incubation with MEMM (50, 100, and 150 *μ*g/mL), morphological modifications of HT29 cells were observed at 100 and 150 *μ*g/mL in comparison to the control untreated cells (Figure 2). Untreated cells were seen to possess normal morphology. Instead, exposure of HT29 cells to 100 and 150 *μ*g/mL MEMM was found to lead to retraction and rounding of cells. In addition, some sensitive cells were found to detach from the surface.

#### 3.6.5. Acridine Orange/Propidium Iodide Staining

Viable HT29 cells were seen with intact DNA, round-shaped nucleus, and green nuclei, whereas the early apoptotic cells were indicated by green, fragmented nucleus. On the contrary, the cells that were undergoing the late apoptotic or necrotic phase were stained orange and red. It is clear from Figure 2 that an increase in the concentration of MEMM (50, 100, and 150 *μ*g/mL) leads to a decrease in the number of viable HT29 cancer cells. Moreover, apoptotic cells also exhibited several other characteristics such as plasma membrane blebbing, nuclear shrinking, and fragmentation.

In addition, the percentage of viable, apoptotic, and necrotic cells was also quantified and presented in Table 7. AO stained early apoptotic cells green, whereas PI stained late apoptotic and necrotic cells. The percentage of viable HT29 cells was decreased once incubated with MEMM. Apoptotic and necrotic cells, however, were increased after 72 h.

#### 3.6.6. UHPLC-ESI-HRMS Profile of MEMM

**Figure 3:**
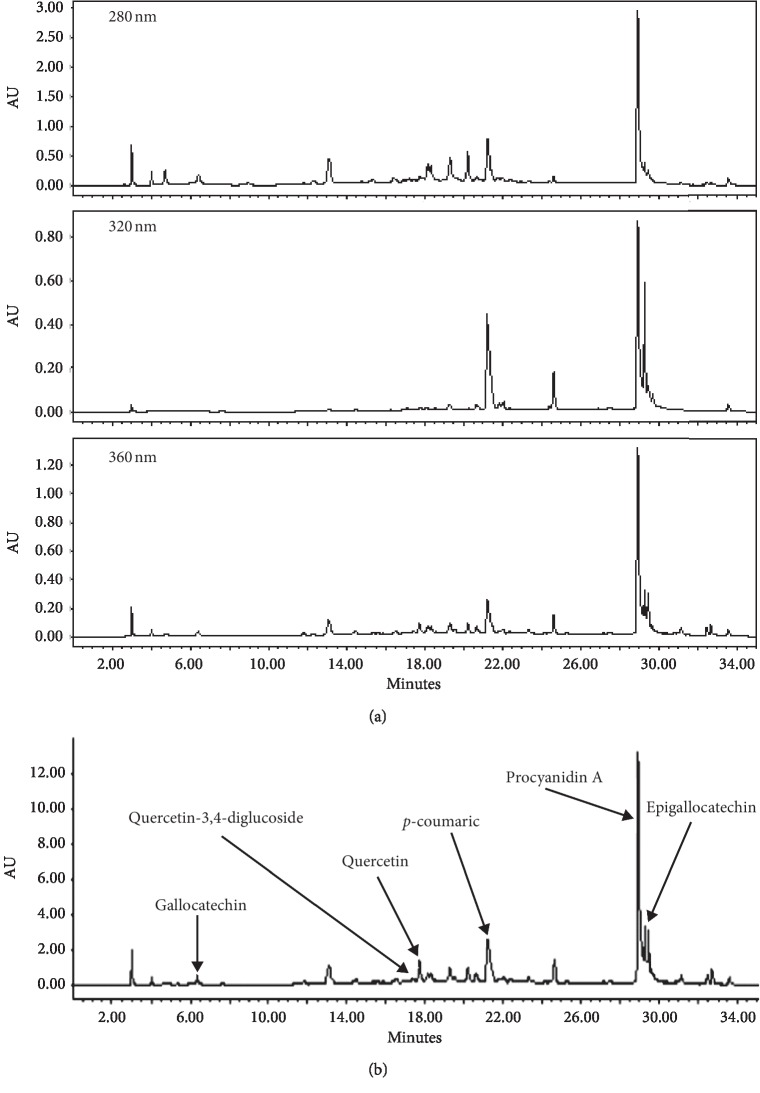
(a) UHPLC-ESI profile of MEMM at various wavelengths (280, 320, and 360 nm). (b) Identification of phytoconstituents of MEMM using the UHPLC-ESI analysis at 360 nm.

MEMM was subjected to the reversed-phase UHPLC-ESI-HRMS analysis to determine its phytochemical constituents using a gradient mobile phase comprising 0.1% aqueous formic acid and 0.1% acetonitrile. This condition will allow for a comprehensive elution of plant analytes within 35 min. Thoroughness in identification was due in part to a higher sensitivity of the UHPLC-MS and processing Xcalibur software. Assignments of metabolites were carried out by comparing the retention time, MS data (accurate mass, isotopic distribution, and fragmentation pattern in the negative ion mode) of the peaks detected with those of the compounds detected in the literature or database. Identification of bioactive compounds was confirmed using standard compounds whenever obtainable in-house. The chromatogram profile of MEMM was obtained at three different wavelengths (250, 320, and 360 nm) (Figure 3(a)).  Figure 3(b) shows some of the phytoconstituents identified in MEMM, which include gallocatechin, quercetin-3,4-diglucoside, quercetin, *p*-coumaric acid, procyanidin A, and epigallocatechin.

## 4. Discussion

For centuries, plant-based natural products have been used throughout the world in the treatment of various disorders. In an attempt to screen for any pharmacological potential, these natural products either in the form of extract, fraction, or compound is usually subjected to the initial evaluation on their toxic characteristics. Despite the presence of various reports on pharmacological potentials of *M. malabathricum* as cited by Mohd. Joffry et al. [4], no thorough knowledge concerning the chronic toxicology of this well-known herb has been published. It is also worth mentioning that the traditional use of any medicinal plant does not guarantee the safety of such a plant for prolonged consumption. Thus, there is a need to obtain data from various models of toxicity studies such as the acute, subacute, subchronic, and, if possible, chronic toxicity on any medicinal plant so as to raise the certainty in its safety to humans especially when the plant has been considered to be developed as pharmaceutical products [17]. To achieve this, it is a crucial step to decide on suitable tests and dosage procedures that will show a sufficient margin of exposure in establishing human safety. Previously, MEMM has been reported to be safe when assessed using the acute toxicity assay. Using the OECD Test Guideline No. 420, the extract, at 5000 mg/kg, was found to cause no sign of toxicity on the tested rats following the 14 days of observation suggesting that the extract has a lethal dose (LD_50_) value greater than 5000 mg/kg and that no further acute testing should be conducted [18]. In addition, Roopashree et al. [19] stated that the limit test method should not be used primarily as a means to determine the exact LD_50_ value, but it should be a means to classify the crude plant extract as being safe or nontoxic depending on the expectation at which dose level the animals are anticipated to survive. Based on the OECD recommendation on chemical labeling and classification of acute systemic toxicity, MEMM can be consigned a class 5 status (LD_50_ > 5000 mg/kg), which refers to the lowest toxicity class. In line with this recommendation, Erhirhie et al. [20] also stated that orally administered compounds with LD_50_ > 5000 mg/kg are considered to be safe or essentially nontoxic.

Since MEMM did not show any sign of toxic effects in the acute toxicity study, additional assessment needs to be carried out to evaluate the subacute (28-day consumption) and subchronic (90-day consumption) toxicities of MEMM in rats to establish the complete toxicity data of MEMM. Toxicological assessments after repeated exposures are required by regulatory agencies to distinguish the toxicological profile of any substance [10].

Subacute and subchronic investigations measure the unwanted effects of frequent or constant exposure of compounds/extracts over a fraction of the average life period of experimental animals, such as rats. Several objectives could be achieved through these studies such as (i) exclusive information on target organ toxicity, (ii) discovery of no observable adverse effect level, and (iii) determination of appropriate dose regimens for longer-term studies [18]. Subsequently, there is inadequate toxicological information in the literature to support and ensure *M. malabatricum* safe use, which triggered the present study with the hope of establishing the subacute and subchronic toxicity profiles of MEMM. To the best of our knowledge, this study reported for the first time on the absence of subacute and subchronic toxicity of MEMM in adult rats.

A considerable decline in food and water intake, which indicates loss of appetite, will lead to a decrease in body weight due to interruptions in the metabolisms of carbohydrate, protein, or fat [21]. Interestingly, the food and water intake was not altered in the group receiving MEMM throughout the 28- or 90-day treatment periods in comparison to the control group, indicating that the extract failed to induce any changes in the metabolisms of carbohydrate, protein, or fat in those rats. Moreover, Yuet Ping [22] also stated that any extract, at higher doses, can be metabolized to a toxic end-product that might hinder the gastric role and decreased food conversion competency. Interestingly, this study also revealed that MEMM did not interfere with the weight gain and appetite stability as seen in the control group that is constantly provided with food and water ad libitum.

Several advantages can be drawn from the findings related to the weighed organs in toxicity studies, which includes (i) the organs sensitivity to acute injury, predict toxicity, physiologic perturbations, and enzyme induction, (ii) the organs are regularly used as target organs of toxicity, (iii) the toxicity effects associate well with histopathological changes, (iv) there is a slight interanimal changeability, and (v) historical control range data are available [23]. Moreover, Mirza and Pancha [24] claimed that the relative organ weights usually measured in toxicity investigations are comparatively sensitive markers for the specific organs and, subsequently, characterize toxicity as substantial changes detected in the particular organs. The outcomes of the present study demonstrated that these essential organs were neither negatively affected nor exerted clinical signs for toxicity throughout the treatment. Thus, it is concluded that MEMM is nontoxic to the analysed organs. Macroscopic observations also further supported the above conclusion as pretreatment with MEMM did not cause changes in colour or presence of hypertrophy on the harvested organs. Hypertrophy of organs is an immediate sign of toxicity following the exposure to a biological or chemical substance. Microscopically, all organs that received MEMM showed no changes in the architecture of cells with any pathologies documented during the histological analysis when viewed under the light microscope.

Estimation of haematological parameters can be utilized to verify the level of detrimental outcomes of compounds/extracts on the blood of tested animals. In addition, such investigation is pertinent to risk assessment as alterations in the haematological parameters have significant prognostic importance for human toxicity when the data are interpreted from animal studies [25]. Following the haematological analysis, MEMM was found to cause no significant changes in the level of several parameters measured. A normal haematological profile observed in MEMM-treated groups when compared to the control group further justified the nontoxic nature of MEMM [26].

Another important indicator of toxicity can be obtained by studying the liver following the administration of test substance. According to Zhang et al. [27], it is important to perform the liver and kidney function analyses during the toxicity assessment of compounds/extracts as data from both analyses are essential for determining the survival of an organism. The estimation of any substance level in the blood can help to facilitate in the early detection of liver injury. Other than the blood parameters, some biochemical markers (i.e., ALT, AST, and ALP) and total bilirubin (TBil) can be used to diagnose a liver injury. A number of enzymes, produced and generally found in the hepatocytes cells of the liver, are involved in the modulation of various chemical reactions in the body. Nevertheless, if the liver is injured or harmed, these enzymes will leak into the blood circulation resulting in the rise of liver enzymes' level, which can be considered as a significant sign of liver toxicity. Meanwhile, a rise in both the total and conjugated bilirubin levels could be used as a measure to determine the overall liver function. An increase in the levels of ALT or AST in combination with an increase to more than double the normal upper level of bilirubin is regarded as a warning sign for hepatotoxicity [28]. Principally, acute or chronic injury to the liver will eventually result in an increase in serum concentrations of AST and ALT. ALT, which is found mainly in the liver, has always been the most commonly relied biomarker of hepatotoxicity and its estimation is considered as a more specific test for detecting liver malfunctions and might indicate hepatocellular necrosis. On the contrary, AST, which is found in the liver as well as in the other organs (i.e., heart, brain, muscle, and kidney) and also assists in spotting hepatocellular necrosis, is regarded as a non-specific biomarker enzyme for liver injury since its serum level elevation can also indicate malfunctions in those other organs. The levels of ALT and AST or together with TBil in rodents and nonrodents are mostly commended for the evaluation of hepatocellular injury in nonclinical investigations. Furthermore, histopathological observations also allow confirmation of hepatotoxicity. With regard to renal dysfunction, concurrent determination on the level of urea, creatinine, and uric acid could be performed and if their normal levels were detected, it is conceivable to suggest the absence of renal problems [29]. In this study, the levels of urea, creatinine, and uric acid in MEMM-treated groups did not differ significantly in comparison to the control group, indicating a normal renal function. Bilirubin, which is an endogenous anion normally present in the blood in small quantities as a result of the normal degradation of haemoglobin, is removed from the liver via the bile system. However, injury to the hepatocytes results in the liver inability to excrete bilirubin in the usual manner, thus increasing the level of bilirubin in the blood and extracellular fluid. Due to this, bilirubin is also classified as a biomarker of hepatobiliary injury and often measured together during the hepatoprotective study. However, in the present study, MEMM was found to cause no change in ALT, AST, and TBil levels. These observations indicated that MEMM did not cause liver damage following the oral administration. With regard to the kidney function, there were also insignificant changes in urea, creatinine, and uric acid levels following the subchronic oral administration of MEMM into rats when compared to the control group. This statement is also concurrent with the present histopathological findings of the kidney tissue, which showed normal architecture, as seen in the control group. Thus, the liver and renal function data supported by their histological findings suggest the nontoxic nature of MEMM.

Although the level of ALP is not considered important in the determination of liver injury, the significant level of ALP detected in the present subchronic study need to be discussed. ALP, which is predominantly found in the cells that line the biliary ducts of the liver, is also found in other organs (i.e., bone, placenta, kidney, and intestine) and removed in the bile. Its serum level may be raised of the normal value if bile excretion is hindered by liver injury due to the congestion or obstruction of the biliary tract (also known as cholestasis). Other than that, the level of ALP, an enzyme that transports metabolites across cell membranes, can also be elevated due to the presence of liver and bone diseases despite the fact that ALP may originate from other tissues, such as the placenta, kidneys, or intestines or from leukocytes [30]. In the present study, there was a significant increase in the level of ALP following pretreatment with MEMM, which was not accompanied by increase in ALT and AST levels, suggesting that the increase in ALP level was not associated primarily with hepatocellular injury [31]. Although cholestasis enhances the synthesis and release of ALP and accumulating bile salts increase its release from the cell surface, their presence can be ruled out as the microscopic examination demonstrated the normal architecture of the liver tissue at all doses of MEMM tested. Several reports have demonstrated the ability of plants' extracts to affect the level of ALP either at the tissue (i.e., liver) or serum level [32, 33]. According to the report by Omage et al. [32], the oral administration of aqueous or ethanol extracts of *Acalypha wilkesiana* leaves significantly changed the level of serum ALP, ALT, and AST when measured at different day intervals (0, 7, 14, and 21 days). Based on their reports, the aqueous extract caused significant reduction in the serum ALP level at day 7 and day 21, whereas the ethanol extract caused significant increase in the serum ALT level only at day 14. As for the serum AST level, the aqueous extract caused significant reduction in the AST level at day 7 and day 21 with significant increase in the AST level seen only at day 14. In comparison, the ethanol extract caused reduction in the serum level of AST at all intervals measured with the significant reduction observed only at day 21. With regard to the serum ALP level, the aqueous extract caused significant increase in the ALP level at day 7 and day 21, while for the ethanol extract, significant increase in the level of serum ALP was observed only at day 21. In comparison to the report made by Omage et al. [32], MEMM did not significantly change or affect the levels of serum ALT and AST, thus suggesting that the increase in the serum ALP level did not directly relate to the MEMM ability to induce liver injury following the oral administration. On the contrary, Yakubu et al. [33] reported on the ability of ethanolic extract of *Khaya senegalensis* stem bark to increase the levels of liver tissue ALP without affecting the level of serum ALP when measured at day 6 and day 18 in comparison to the control group, and these findings were also observed for the liver tissue and serum levels of AST. However, the liver tissue level of ALT, which was decreased at day 6 and increased at day 18, was also accompanied by the increased in the serum ALT level at day 6, but not day 18. According to Yakubu et al. [33], the significant increase in the liver ALP activity following the administration of the plant extract may be due to increased functional activity of the liver, probably leading to de novo synthesis of the enzyme molecules. Such excess level of serum ALP, which was accompanied by no concomitant increase in the serum level of ALT, AST, as well a TBil, may suggest that the integrity of the liver plasma membrane was not compromised following the administration of the plant extract. However, since the ALP hydrolyses phosphate monoesters, which play role in the facilitation of the transfer of metabolites across the cell membrane, the subchronic and chronic use of *M. malabathricum* leaves need to be carried out with caution. Furthermore, the fact that the effect of MEMM on the serum ALP level was observed only in the subchronic study could also be associated with the ALP's longer half-life, which caused the enzyme serum level to decrease slowly after resolution and make the enzyme stay in the circulation longer in comparison to ALT and AST [34].

Chemotherapy has been successfully used in the treatment of several tumors such as testicular cancer and certain leukaemia's. However, its achievement against common epithelial tumors of the breast, colon, and lung has been less than impressive [35]. Preferably, chemotherapeutic agents should exclusively target just neoplastic cells and should reduce tumor burden by stimulating cytotoxic effects without injuring the normal cells. However, the efficacy of chemotherapy has experienced a variety of confounding factors including systemic toxicity, which is attributable to the unspecific action, instantaneous metabolism of drug, and both intrinsic and acquired drug resistance [30]. Due to these factors and worsened by other issues such as ineffective therapeutic strategies to control and treat colon cancer, the high financial burden incurred on the patients and their families as well as the nations have demanded the look for novel remedies largely from the natural product sources [36]. This might explain the increase in awareness in the usage of natural products as an alternative approach to effectively control cancers in recent years [37].

One of the most important methods for evaluating anticancer properties of any extract/compound is the cytotoxicity test, which uses cancer cells *in vitro* to watch the cancer cell growth, reproduction, and morphological effects upon exposure to the extract/compound [38]. Cytotoxicity has a series of advantages such as it is modest, precipitous, highly sensitive, and can protect the animals from toxicity [38]. With the constant progress in cytotoxicity testing, various procedures, including discovery of cell injury by observing the cells' morphological changes, determining the mode of cells damage, quantifying the cells' growth and metabolic properties, have emerged and had progressively been expanded from qualitative approaches to quantitative evaluations [39].

One of the mechanisms of anticancer is apoptosis, which is a very organized physiological machinery to abolish damaged or abnormal cells [40]. Apoptosis, which is a programmed cell death, is a smart screening endpoint in the discovery and development of novel anticancer drugs. An extensive range of natural substances present in plants has been documented to possess the capacity to cause apoptosis in numerous tumor cells of human origin [41, 42]. MEMM possessed high content of phenolic compounds [8] and exerts many pharmacological and biological activities including antioxidative [8], anti-inflammatory [43], and antiproliferative activities [44]. Various bioactive compounds have been isolated from MEMM, such as ursolic acid, 2-hydroxyursolic acid, asiatic acid, gallic acid, *p*-hydroxybenzoic acid, kaempferol, kaempferol-3-O-(2″,6″ di-*O*-*p*-*trans*-coumaroyl)-*β*-glucoside, *α*-amyrin, uvaol, quercetin, quercitrin, and rutin [4]. Of these, ursolic acid [45], asiatic acid [46], kaempferol [47], quercetin [48], and rutin [49] have been reported to exert anticolon cancer activity against the HT29 cancer cell line. In the recent report, MEMM was analysed using the UHPLC-ESI procedure and revealed the presence of caffeic acid, chlorogenic acid, *p*-coumaric acid, gallocatechin, epigallocatechin, catechin, quercetin, quercetin-3-*O*-glucoside, and hesperidin [50]. Of these, caffeic acid [51], quercetin, and *p*-coumaric [52] have been reported to exert anticolon cancer activity against the HT29 cancer cell line. Thus, it is reasonable to propose that the observed anticolon cancer activity of MEMM involves, in part, the synergistic action of those bioactive compounds.

## 5. Conclusion

In conclusion, *M. malabathricum* leaves, in the form of MEMM, did not exert any signs of toxic effects on rats with regard to their behaviour, body weight, haematological and biochemical parameters, and relative organs weight following the subacute (28 days) or subchronic (90 days) oral administration of the extract. Hence, no observed adverse-effect level (NOAEL) was detected, and NOAEL for this extract has been determined to be greater than 500 mg/kg. MEMM also showed cytotoxic activity against the HT29 colon cancer cells partly via apoptosis and possibly through the synergistic action of several flavonoids presence in the extract.

## Figures and Tables

**Table 1:** Relative organ weight of Sprague-Dawley rats following the 28-day oral pretreatment with vehicle (Tween 80) or MEMM in the subacute toxicity study.

Organs	Male			Female		
	Control	Treatment		Control	Treatment	
	Tween 80	MEMM (mg/kg)		Tween 80	MEMM (mg/kg)	
	**—**	500	1000	—	500	1000
Spleen	0.62 ± 0.09	0.53 ± 0.13	0.61 ± 0.17	0.71 ± 0.03	0.63 ± 0.09	0.39 ± 0.03
Heart	0.47 ± 0.04	0.42 ± 0.03	0.46 ± 0.03	0.42 ± 0.05	0.39 ± 0.03	0.42 ± 0.03
Liver	5.35 ± 0.19	5.14 ± 0.44	5.58 ± 0.30	4.97 ± 0.44	5.10 ± 0.27	4.69 ± 0.28
Kidney	1.46 ± 0.06	1.41 ± 0.07	1.55 ± 0.05	1.29 ± 0.12	1.20 ± 0.11	1.08 ± 0.02
Stomach	0.95 ± 0.14	0.78 ± 0.07	0.80 ± 0.06	0.87 ± 0.13	0.71 ± 0.07	0.90 ± 0.04
Lung	0.85 ± 0.04	0.88 ± 0.14	0.99 ± 0.08	0.87 ± 0.04	1.20 ± 0.27	0.77 ± 0.04

Values are expressed as means ± SEM. Groups (*n* = 6): control (treated with Tween 80); treatments (treated with 500 mg/kg MEMM and 1000 mg/kg MEMM).

**Table 2:** Relative organ weight of Sprague-Dawley rats following the 90-day oral pretreatment with vehicle (Tween 80) or MEMM in the subchronic toxicity study.

Organs	Male			
	Control	Treatment		
	Tween 80	MEMM (mg/kg)		
	**—**	50	250	500
Spleen	0.21 ± 0.04	0.17 ± 0.01	0.20 ± 0.02	0.16 ± 0.01
Liver	2.76 ± 0.35	3.76 ± 0.07	2.93 ± 0.25	2.71 ± 0.12
Heart	0.35 ± 0.03	0.33 ± 0.01	0.34 ± 0.02	0.29 ± 0.02
Kidney	0.74 ± 0.06	0.64 ± 0.02	0.72 ± 0.03	0.61 ± 0.01
Lung	0.78 ± 0.07	0.71 ± 0.04	0.95 ± 0.13	0.90 ± 0.11
Colon	0.84 ± 0.13	0.77 ± 0.07	0.81 ± 0.07	0.70 ± 0.03

Values are expressed as means ± SEM. Groups (*n* = 6): control (treated with Tween 80); treatments (treated with 50 mg/kg MEMM, 250 mg/kg MEMM, and 1000 mg/kg MEMM).

**Table 3:** Haematological profiles of Sprague-Dawley rats following the 28-day oral pretreatment with vehicle (Tween 80) or MEMM in the subacute toxicity study.

Haematological parameters	Male			Female		
	Control	Treatment		Control	Treatment	
	Tween 80	MEMM (mg/kg)		Tween 80	MEMM (mg/kg)	
	**—**	500	1000	—	500	1000
RBC (×10^12^/L)	8.36 ± 0.22	8.42 ± 0.31	8.66 ± 0.77	7.73 ± 0.93	7.89 ± 0.83	9.46 ± 0.47
WBC (×10^9^/L)	3.74 ± 0.41	3.02 ± 0.81	4.86 ± 0.93	4.83 ± 0.51	6.30 ± 1.62	3.62 ± 0.70
Hb (g/L)	138.40 ± 3.06	129.40 ± 5.04	122.20 ± 8.41	125.71 ± 11.44	108.40 ± 13.11	134.00 ± 12.36
PCV (L/L)	0.36 ± 0.01	0.33 ± 0.01	0.31 ± 0.02	0.41 ± 0.10	0.27 ± 0.03	0.32 ± 0.02
MCV (fL)	43.40 ± 2.51	39.00 ± 1.05	35.80 ± 2.65	37.21 ± 0.62	34.60 ± 0.87	34.20 ± 1.99
MCHC (g/L)	382.80 ± 7.81	395.20 ± 6.49	401.20 ± 10.85	388.67 ± 22.21	393.80 ± 18.00	413.20 ± 16.46
Neutrophils (×10^9^/L)	10.80 ± 1.20	10.60 ± 1.94	9.50 ± 2.5	12.61 ± 1.27	14.20 ± 2.60	16.00 ± 2.52
Lymphocytes (×10^9^/L)	71.60 ± 1.60	79.20 ± 3.40^∗^	81.25 ± 3.84	68.77 ± 5.34	57.00 ± 4.67	73.33 ± 2.73
Monocytes (×10^9^/L)	5.00 ± 0.55	6.40 ± 1.44	5.08 ± 0.41	5.41 ± 0.52	6.40 ± 1.03	4.67 ± 0.33
Eosinophils (×10^9^/L)	3.40 ± 0.75	2.40 ± 0.24	2.75 ± 0.48	3.22 ± 0.43	4.20 ± 1.72	4.00 ± 1.16

Values are expressed as means ± SEM. Groups (*n* = 6): control (treated with Tween 80); treatments (treated with 500 mg/kg MEMM and 1000 mg/kg MEMM).

**Table 4:** Haematological profiles of Sprague-Dawley rats following the 90-day oral pretreatment with vehicle (Tween 80) or MEMM in the subchronic toxicity study.

Haematological parameters	Male			
	Control	Treatment		
	Tween 80	MEMM (mg/kg)		
	**—**	50	500	1000
RBC (×10^12^/L)	8.15 ± 0.32	8.25 ± 0.17	6.98 ± 0.91	6.61 ± 0.73
WBC (×10^9^/L)	153.30 ± 9.42	155.80 ± 2.50	133.31 ± 15.80	132.80 ± 14.73
Hb (g/L)	0.40 ± 0.02	0.41 ± 0.01 0	0.34 ± 0.04	0.35 ± 0.04
PCV (L/L)	49.25 ± 0.75	49.75 ± 1.32	49.25 ± 1.60	52.25 ± 0.63
MCV (fL)	383.00 ± 11.71	380.80 ± 10.04	393.00 ± 6.55	385.30 ± 6.30
MCHC (g/L)	8.44 ± 1.86	7.85 ± 0.69	11.08 ± 3.12	8.30 ± 1.87
Neutrophils (×10^9^/L)	2.51 ± 0.50	3.48 ± 0.58	3.03 ± 0.92	3.53 ± 0.83
Lymphocytes (×10^9^/L)	4.89 ± 1.16	3.60 ± 0.20	6.42 ± 1.24	3.92 ± 0.93
Monocytes (×10^9^/L)	0.55 ± 0.14	0.46 ± 0.08	0.61 ± 0.14	0.53 ± 0.17
Eosinophils (×10^9^/L)	0.32 ± 0.08	0.32 ± 0.05	0.35 ± 0.04	0.32 ± 0.02

Values a reexpressed as means ± SEM. Groups (*n* = 6): control (treated with Tween 80); treatments (treated with 50 mg/kg MEMM, 250 mg/kg MEMM, and 1000 mg/kg MEMM)

**Table 5:** Biochemical profiles of liver of Sprague-Dawley rats following the 28-day oral pretreatment with vehicle (Tween 80) or MEMM in the subacute toxicity study.

Serum liver parameters	Male			Female		
	Control	Treatment		Control	Treatment	
	Tween 80	MEMM (mg/kg)		Tween 80	MEMM (mg/kg)	
	**—**	500	1000	—	500	1000
ALT (U/L)	83.44 ± 21.06	107.80 ± 33.25	138.10 ± 33.18	90.71 ± 10.18	240.60 ± 77.36	94.18 ± 31.23
ALP (U/L)	154.80 ± 23.57	153.80 ± 22.98	98.60 ± 9.42	122.26 ± 14.35	103.80 ± 8.02	115.80 ± 12.48
AST (U/L)	352.40 ± 78.28	342.30 ± 65.90	227.73 ± 55.90	381.77 ± 48.37	332.04 ± 36.80	344.71 ± 38.70
TBil (*μ*mol/L)	0.80 ± 0.08	0.62 ± 0.09	0.76 ± 0.04	0.41 ± 0.07	0.28 ± 0.07	0.24 ± 0.09
Creatinine (*μ*mol/L)	33.60 ± 1.36	41.40 ± 8.79	38.40 ± 6.56	36.57 ± 8.21	47.00 ± 8.01	32.40 ± 5.79
Urea (mmol/L)	8.64 ± 0.58	10.73 ± 0.67	10.83 ± 0.60	9.52 ± 0.94	11.95 ± 0.19	6.97 ± 0.87

Values are expressed as means ± SEM. Groups (*n* = 6): control (treated with Tween 80); treatments (treated with 500 mg/kg MEMM and 1000 mg/kg MEMM).

**Table 6:** Biochemical profiles of liver of Sprague-Dawley rats following the 90-day oral pretreatment with vehicle (Tween 80) or MEMM in the subchronic toxicity study.

Serum liver parameters	Male			
	Control	Treatment		
	Tween 80	MEMM (mg/kg)		
	**—**	50	500	1000
ALT (U/L)	52.58 ± 4.62	57.65 ± 10.89	55.88 ± 5.78	59.08 ± 7.10
ALP (U/L)	136.80 ± 25.87	151.50 ± 15.88	224.80 ± 37.52^*∗*^	236.50 ± 16.83^*∗*^
AST (U/L)	170.30 ± 16.42	235.60 ± 48.06	224.10 ± 49.70	223.60 ± 45.40
TBil (*μ*mol/L)	1.58 ± 0.31	1.36 ± 0.25	1.43 ± 0.13	1.47 ± 0.18
Creatinine (*μ*mol/L)	61.00 ± 2.12	57.75 ± 2.60	54.75 ± 1.80	58.50 ± 2.50
Urea (mmol/L)	7.15 ± 0.93	7.33 ± 1.07	5.43 ± 0.30	5.75 ± 0.30

Values are expressed as means ± SEM. Significant different as compared to control, *p* < 0.05. Groups (*n* = 6): control (treated with Tween 80); treatments (treated with 50 mg/kg MEMM, 250 mg/kg MEMM, and 1000 mg/kg MEMM)

**Table 7:** Quantitative approach of MEMM and AQMM towards human colon cancer cell line (HT29) once incubated at different concentrations after 72 h.

Cells	MEMM (mg/mL)		
	50	100	150
Viable (%)	36	26	6
Apoptotic (%)	50	59	72
Necrotic (%)	14	16	22

## Data Availability

The supporting materials can be obtained upon request via email to the corresponding author.

## Abbreviations

MEMM: Methanol extract of *Melastoma malabathricum*
OECD: Organisation for Economic Co-operation and Development
MTT: 3-(4,5-Dimethylthiazol-2-yl)-2,5-diphenyltetrazolium bromide
ALP: Alkaline phosphatase
UPM: Universiti Putra Malaysia
IBS: Institute of Bioscience
IACUC: Institute Animal Care and Use Committee
RBC: Red blood count
Hb: Haemoglobin
MCHC: Mean corpuscular haemoglobin concentration
MCV: Mean corpuscular volume
PCV: Packed cell volume
WBC: White blood cell
ALT: Alanine aminotransferase
AST: Aspartate aminotransferase
Crea: Creatinine
TBil: Total bilirubin
ATCC: American Type Culture Collection
RPMI: Roswell Park Memorial Institute 1640
FBS: Fetal bovine serum
Pen-Strep: Penicillin-streptomycin
CO_2_: Carbon dioxide
DMSO: Dimethyl sulfoxide
PBS: Phosphate buffer saline
AO: Acridine orange
PI: Propidium iodide
ANOVA: Analysis of Variance
LD: Lethal dose
NOAEL: Observed-adverse-effect level.
